# The Effect of Sevoflurane Anesthesia on the Biomarkers of Neural Injury in the Prefrontal Cortex of Aged Marmosets

**DOI:** 10.3389/fnagi.2022.918640

**Published:** 2022-06-30

**Authors:** Yanyong Cheng, Lingling Shi, Haoli Mao, Zhenyu Xue, Siyu Liu, Zilong Qiu, Lei Zhang, Hong Jiang

**Affiliations:** ^1^Department of Anesthesiology, Shanghai Ninth People's Hospital, Shanghai Jiao Tong University School of Medicine, Shanghai, China; ^2^State Key Laboratory of Neuroscience, CAS Center for Excellence in Brain Science and Intelligence Technology, Shanghai Center for Brain Science and Brain-Inspired Intelligence Technology, Institute of Neuroscience, Chinese Academy of Sciences, University of Chinese Academy of Sciences, Shanghai, China

**Keywords:** general anesthetic, sevoflurane, neural injury, cytokine, primate

## Abstract

**Background:**

Surgery under general anesthesia leads to neural injury, especially in older patients. Sevoflurane anesthesia without surgery for 2 h does not induce neural injury, however, whether prolonger sevoflurane anesthesia without surgery has the same consequence is still unknown.

**Methods:**

In the present study, aged marmosets were exposed to a clinical concentration of sevoflurane (1.5–2%) for 6 h to access the effects of prolonged sevoflurane anesthesia on the levels of interleukin-6 (IL-6) and tumor necrosis factor-α (TNF-α), Caspase3 activity and myelin formation in the brain.

**Results:**

Sevoflurane anesthesia did not alter the expression of IL-6 (120.1 ± 2.21 vs. 120.8 ± 2.25, *p* = 0.74), TNF-α (189.3 ± 31.35 vs. 218.7 ± 21.47, *p* = 0.25) and Caspase3 (57.35 ± 1.54 vs. 58.67 ± 1.19, *p* = 0.53) in the prefrontal cortex (PFC) of aged marmosets. The amount of MBP expression (60.99 ± 6.21 vs. 58.91 ± 2.71, *p* = 0.77) did not change following sevoflurane exposure.

**Conclusion:**

Sevoflurane anesthesia did not increase the levels of IL-6 and TNF-α, activated the the expression of Caspase3, and induced myelination deficits in the PFC of aged marmosets.

## Introduction

The increase of interleukin-6 (IL-6) and tumor necrosis factor-α (TNF-α) during the perioperative period has been associated with postoperative complications and prolonged hospitalization in patients after surgery (O'bryan et al., 2022). However, the contribution of general anesthesia and surgery can hardly be differentiated in clinical settings, as patients receive general anesthesia only when they undergo surgery. In a recent prospective cohort study, 59 elderly volunteers received sevoflurane general anesthesia without surgery for 2 h, and the result showed no increase in IL-6 and TNF-α in their blood (Deiner et al., 2020). However, due to the more complex disease presentation in the elderly, surgical procedures take longer than in younger people. A previous study revealed that sevoflurane exposure for 5 h in aged rats induced apoptosis of the neurons, which led to learning and memory deficits (Chen et al., 2013). We consider whether IL-6 and TNF-α, as well as other injury markers such as the markers of apoptosis and myelination deficits, are altered in the brain with prolonged sevoflurane general anesthesia. As it is not feasible to take these measurements in the human brain, Marmosets (*Callithrix jacchus*) are increasingly used as an alternative primate model in biomedical research because their genetic and neuroanatomical features are closer to those of humans. However, no study identified the effects of sevoflurane anesthesia on inflammation factors and other neuroinjury markers in aged marmosets.

The activation of neuroinflammation and apoptosis are mutually reinforcing. Recent studies have suggested that the inflammation and apoptosis process were not limited to neurons and that it can also be found in non-neuron cells, such as oligodendrocytes (OLs). The myelin sheath which constructed of OLs was concerned in this study simultaneously.

The present study assessed the effects of acute sevoflurane anesthesia, without surgery, on the amounts of IL-6 and TNF-α as well as the markers of apoptosis (Caspase3) and myelination deficits via Elisa, immunohistochemistry, and transmission electron microscopy in the PFC of aged marmosets.

## Methods And Materials

### Marmoset Anesthesia

The processes of the animal study were performed according to the guidelines of the Animal Care Committee of the Center for Excellence in Brain Science and Intelligence Technology (CEBSIT, China) and were approved by the Institutional Animal Care and Use Committee (Protocol number CEBSIT-2021035). Sexually mature common marmosets aged 18–24 months were purchased from CEBSIT. They were considered to be “aged” when they were >8 years old.

Two male and one female marmoset (6 marmosets in total) were involved in each group (the control and anesthesia group). Efforts were made to minimize the number of marmosets. In consideration of the restricted number of marmosets in this study, we did not take the potential sex difference of the anesthesia in marmosets into account. The marmosets received induction (2–4 min) under 6–8% sevoflurane with 100% oxygen. For the maintenance of general anesthesia, then they received 1.5–2% sevoflurane and 100% oxygen without endotracheal intubation for 6 h. Spontaneous respirations were retained during the general anesthesia.

The temperatures of the marmosets were maintained at 37°C by an animal warming placing system (AHM06, Reptizoo, China). The heart rate, electrocardiograph, respiratory rate, peripheral capillary oxygen saturation (SpO_2_), and rectal temperature were monitored by Patient Monitors (BeneVision M12, Mindray, China) (Supplementary Video). The arterial blood gas determinations were monitored by cardiac puncture using a portable clinical analyzer (i-STAT; Abbott Laboratories Inc., East Windsor, NJ, USA) at the end of the anesthetic. An infusion of 5 mL stroke-physiological saline solution was applied every 2 h to prevent dehydration.

For the sampling stage, in the control group, marmosets received high concentrations (6–8%) of anesthetic sevoflurane (about 1 min) for quick entering a sufficient depth of anesthesia. Then they were decapitated and under 5% sevoflurane for about 1–2 min. No body movements and reflexes were observed during the execution. Marmosets in the sevoflurane group were decapitated under 3% sevoflurane anesthesia for 5 min with a complete loss of all reflexes. The PFC of all marmosets was harvested.

### Elisa

Marmoset brain tissues were lysed using RIPA buffer with a protease inhibitor cocktail. The TNF-α or IL-6 standards were re-suspended by adding 500 μL Sample Diluent NS. It was put at room temperature for 10 min and mixed gently. This is the 5,000 pg/mL Stock Standard Solution. Label eight tubes, Standards 1 to 8. We added 320 μL Sample Diluent NS into tube number 1 and 150 μL of Sample Diluent NS into numbers 2 to 8.

TNF-α and IL-6 ELISA kits were used (# ab252354, ab242233, abcam, USA). We added 50 μL of all samples or standard to appropriate wells and then 50 μL of the Antibody Cocktail to each well. Wells were incubated for 1 h at room temperature on a plate shaker which was set to 400 rpm and washed 3 times by Wash Buffer PT. Wash Buffer PT should remain in wells for at least 10 s. We added 100 μL of TMB Development Solution to each well and incubated it for 20 min in the dark on a plate shaker set to 400 rpm. Then we halted reactions with 100 μL of Stop Solution and shacked for 1 min to mix. We determined the optical density of each well using a Fluorescence Plate Reader (Medical Device, San Jose, CA, USA) at 450 nm. Next, we created a standard curve by plotting the average blank control subtracted absorbance value for each standard concentration (y-axis) against the target protein concentration (x-axis) of the standard. We draw the best smooth curve through these points to construct the standard curve by Graphing software. We determined the concentration of the target protein in the sample by interpolating the blank control subtracted absorbance values against the standard curve. The resulting value was multiplied by the appropriate sample dilution factor to obtain the concentration of the target protein in the sample.

### Immunohistochemistry

After prolonged sevoflurane inhalation, marmosets were euthanized and brains were immersed in 4% paraformaldehyde fixed over days. Prefrontal cortex paraffin slices were deparaffinized with xylene and washed with anhydrous ethanol, 95 and 75% alcohol by volume. After being blocked with 10% donkey serum albumen and 0.3% Triton for 2 h, the slices were stained with primary antibodies and secondary antibodies (anti-MBP #NB600-717, 1:200 dilution, Novus Biologicals, USA; anti-Caspase 3 #NB600-1235,1:200 dilution, Novus Biologicals, USA) at 4°C overnight; Goat Anti-Rabbit IgG H&L (#ab150077, 1:1000 dilution, abcam, USA) for 2 h at room temperature. Nuclei were visualized with DAPI and images were taken with upright fluorescence microscopy (Nikon, Japan). Antibody-positive cells were counted by manual counting. The number of target channels was normalized to DAPI. Three animals for each group and 5 to 7 brain slices from each animal were used. We calculated the number of antibody-positive cells within a certain area (2,500 μm^2^) of each slice. Each contained at least 100 cells.

### Transmission Electron Microscopy

Marmosets were euthanized and perfused with PBS for preparing tissue samples for transmission electron microscopy. Issues were fixed in 2.5% glutaraldehyde and then postfixed in 1% osmium tetroxide. They were dehydrated and embedded in araldite resin. Ultrathin sections (60 nm) were stained in uranyl acetate and lead citrate. A transmission electron microscope (H-7650, hitachi, Japan) at 80 kV was used.

### Statistical Analysis

An unpaired *t*-test was used to compare the mean of the two groups, and *p* < 0.05 were considered statistically significant. We used the software Prism 6 (GraphPad, USA) to evaluate all of the data in the studies.

## Results

In the present study, we used marmosets aged 8–9 years to evaluate the contribution of anesthetics to neural injury. Marmosets older than 8 years old were considered aged marmosets (Abbott et al., 2003). The characteristics of the aged marmosets in both the control and sevoflurane anesthesia groups are shown in Supplementary Table 1. Several biomarkers of neural injury were documented in this study from various aspects, such as cytokine, the critical enzyme of apoptosis, and indicator of the myelin sheath.

**Figure 1:**
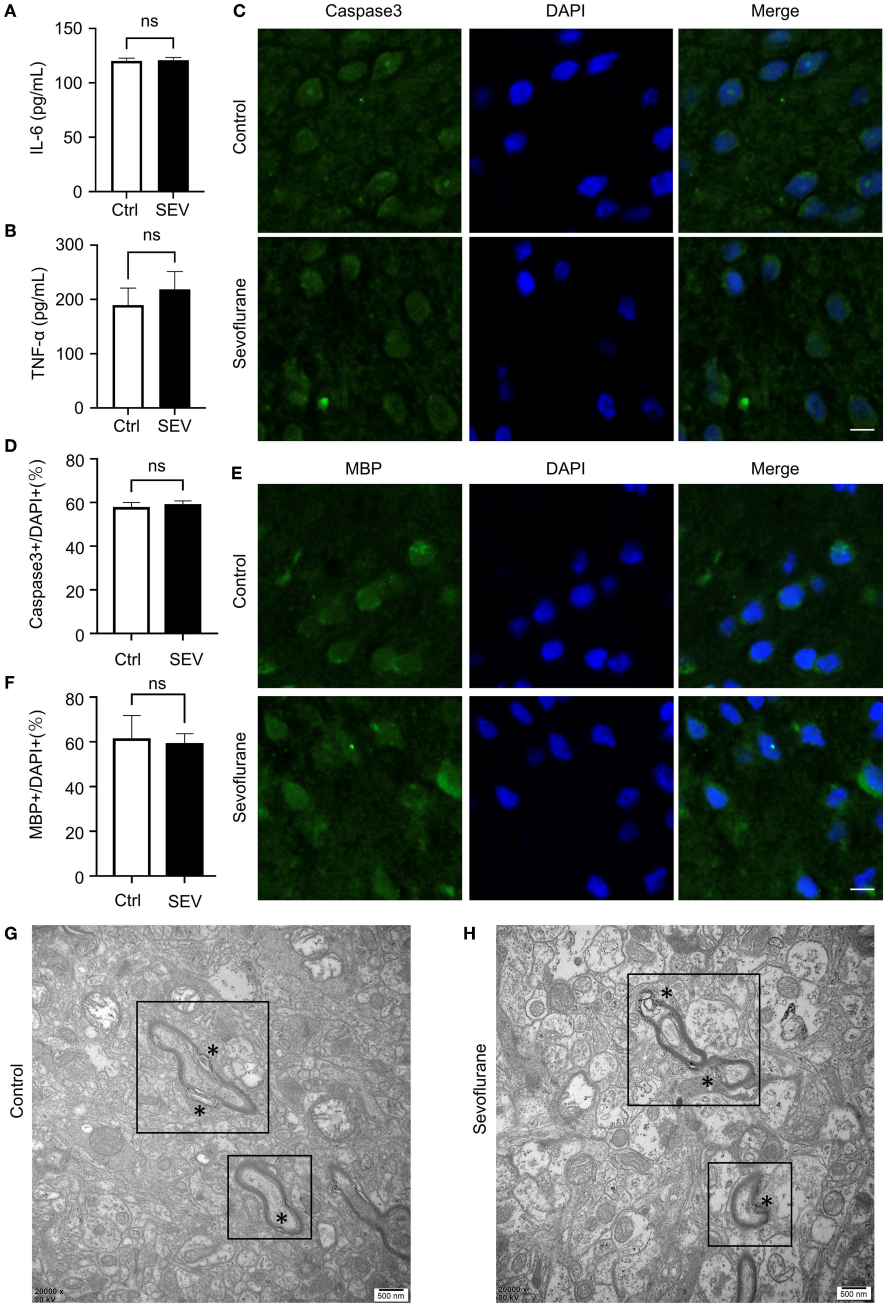
The effect of sevoflurane anesthesia on the biomarkers of neural injury in the prefrontal cortex (PFC) of aged marmosets. **(A,B)** Elisa analysis indicated the amounts of IL-6 (*n* = 3, 120.1 ± 2.21 vs. 120.8 ± 2.25, *p* = 0.74, one-way ANOVA) and TNF-α (*n* = 3, 189.3 ± 31.35 vs. 218.7 ± 21.47, *p* = 0.25, one-way ANOVA) in the PFC of the marmosets in Ctrl and SEV groups. **(C)** Immunofluorescence staining showed that Caspase3 protein level did not alter in aged marmosets' PFC upon sevoflurane exposure as quantified by the number of Caspase3+ cells. The scale bar indicates 50 μm. **(D)** The number of target channels (Caspase3) was normalized to the number of DAPI+ cells in marmosets' PFC. Sevoflurane did not alter Caspase3 expression in marmosets' prefrontal cortical compared to control condition (n = 3, 57.35 ± 1.54 vs. 58.67 ± 1.19, *p* = 0.53, one-way ANOVA). **(E)** Immunofluorescence staining exhibited that MBP protein level did not alter in aged marmosets' PFC upon sevoflurane exposure as quantified by the number of MBP+ cells. The scale bar indicates 50 μm. **(F)** The number of MBP+ cells was normalized to the number of DAPI+ cells in marmosets' PFC. Sevoflurane did not alter MBP expression in marmosets' prefrontal cortical compared to control condition (n = 3, 60.99 ± 6.21 vs. 58.91 ± 2.71, *p* = 0.77, one-way ANOVA). **(G,H)** Electron microscopy showed the loosening and splits (*) in adjacent membrane layers and myelin distortions in both Ctrl group **(G)** and SEV group **(H)**. The myelination deficits in the sevoflurane group looked no more serious than that in the control group. The scale bar indicates 0.5 μm.

We first assessed the effects of sevoflurane without surgery on the level of IL-6 and TNF-α in the PFC of aged marmosets by ELISA. The result suggested that prolonged sevoflurane anesthesia for 6 h without surgery did not alter the expression of IL-6 (120.1 ± 2.21 vs. 120.8 ± 2.25, *p* = 0.74, Figure 1A) and TNF-α (189.3 ± 31.35 vs. 218.7 ± 21.47, *p* = 0.25, Figure 1B) in the PFC of aged marmosets.

Secondly, to investigate whether the activation of Caspase3, a critical enzyme of apoptosis, was affected by sevoflurane anesthesia, immunofluorescence was employed to detect the Caspase3 activation in the PFC of aged marmosets. Caspase3 showed no significant changes in protein levels (57.35 ± 1.54 vs. 58.67 ± 1.19, *p* = 0.53, Figures 1C,D).

Finally, immunofluorescence staining was used to explore the structure and status of the myelin sheath following sevoflurane anesthesia. The expression level of myelin basic protein (MBP) was insusceptible to the sevoflurane exposure (60.99 ± 6.21 vs. 58.91 ± 2.71, *p* = 0.77, Figures 1E,F). The formation of ultrastructure in marmosets' PFC was also illustrated by electron microscopy. Myelin sheath was followed with interest. Although we observed degeneration of the myelin sheath, mainly as lamellae loosening, splits and other myelin distortions, in both groups of aged marmosets, the sevoflurane group did not show significant changes compared with the control group (Figures 1G,H).

## Discussion

To explore whether general anesthesia without surgery induces the process of neural injury (neuroinflammatory, apoptosis, and myelination deficits), we assessed the altered expression of IL-6 and TNF-α, Caspase3 activation, levels of MBP, and the ultrastructure of myelin sheath in the PFC of aged marmosets. The sevoflurane anesthesia with a duration of 6 h was applied based on previous studies (Schenning et al., 2017; Lai et al., 2021). Five to six hours of exposure to inhalation anesthetics were commonly used in the study of perioperative neural disorders. As a result, prolonged sevoflurane anesthesia did not increase the levels of IL-6 and TNF-α, activated the expression of Caspase3, and induced myelination deficits in the PFC of aged marmosets.

The results of the neuroinflammatory process are consistent with previous studies. Isoflurane anesthesia for 6 h did not increase IL-6 and interleukin-1β (IL-1β) in blood, spleen, and hippocampus in 18-month-old mice (Lai et al., 2021). Also, in adult rhesus macaques (16–17 years of age), an 8-hour-exposure to sevoflurane anesthesia did not induce microglial activation (Walters et al., 2019). Consistently, 59 elderly volunteers received sevoflurane general anesthesia without surgery for 2 h, with no increase in the level of IL-6 and TNF-α in their blood (Deiner et al., 2020). Our results from aged marmosets also suggested that sevoflurane anesthesia for 6 h has no impact on the expression of IL-6 and TNF-α. Surgical trauma, but not anesthesia, could be the critical factor in increasing the levels of inflammation factors.

Existing evidence demonstrates increased apoptotic death in the brains of young rhesus macaques by anesthetic sevoflurane (5h) (Rosado-Mendez et al., 2019). Our previous findings also supported the conclusion that sevoflurane exposure induced developmental toxicity to the myelin sheath in young rhesus macaques (Zhang et al., 2019). These findings suggested that anesthesia leads to neuroinflammation and apoptosis in young non-human primates, however, whether sevoflurane anesthesia without surgery causes the same consequence in aged non-human primates is still unknown. Notably, in the present study, sevoflurane did not induce Caspase3 activation and myelination deficits in the PFC of aged marmosets. In general, there was no significant difference in caspase3 in aged marmosets after sevoflurane exposure, while neural apoptosis was found in young rhesus macaques. As brain structure and functions differ with age (Zhao et al., 2019), anesthetic management of the aged requires different approaches compared with that of the young (Purdon et al., 2015). In the young brain, nerve cells are mainly in the process of proliferation and development. The cells can always assist in the maintenance of normal brain function. They rarely undergo apoptosis under normal conditions. Cellular stress or drugs could cause abnormal changes and apoptosis in nerve cells. After sevoflurane exposure, significant differences could be found more easily. While age-related neuronal apoptosis is obvious in the aged brain. After sevoflurane exposure, the process of apoptosis may be accelerated, but not easy to be detected. The immediate injury might be masked over in the original state of chronic age-related injury. The age difference was exactly a complication to the effect of sevoflurane exposure. The different effects of sevoflurane on the age and the young is also a potential direction of study that requires further research.

There were some limitations in our study. Firstly, we harvested the brain tissue immediately after sevoflurane anesthesia, and the myelination deficits may not be reflected at this point. This study only examined acute sevoflurane exposure. If we want to focus on the long-term effect of sevoflurane exposure, the sampling time should be appropriately extended. Secondly, we did not assess enough neuroinflammation and apoptosis factors. These three markers are commonly used and important in neuroscience research. Due to this unusual species, it was difficult to find the appropriate Elisa kits and the antibody for marmosets. The sample size might also be insufficient for this current study. Due to the scarcity and high cost of laboratory primates, efforts were made to minimize the number of marmosets. Though the sample size of three in each group usually satisfies the bottom line, it is not large enough to draw a persuasive conclusion. Here we found this phenomenon and presented some clues of the sevoflurane's effect on aged primates by restricted sample sizes. It is feasible to carry out experiments under the direction of the findings in this preliminary study.

## Data Availability Statement

The raw data supporting the conclusions of this article will be made available by the authors, without undue reservation.

## Ethics Statement

The process of the animal study was performed according to the guidelines of the Animal Care Committee of Center for Excellence in Brain Science and Intelligence Technology (CEBSIT, China) and was approved by the Institutional Animal Care and Use Committee (Protocol number CEBSIT-2021035). The marmosets were purchased from CEBSIT.

## Author Contributions

LZ and HJ: conceptualization. YC, HM, ZX, SL, and LS: methodology. YC, LS, and LZ: writing—original draft preparation. LZ, ZQ, and HJ: writing—review and editing. All authors contributed to the article and approved the submitted version.

## Funding

This work was supported by NSFC Grants (#81970990, #82071177, and #82171173), the Shanghai Jiao Tong University School of Medicine Two-hundred Talent (20191818), the Cross Disciplinary Research Fund of Shanghai Ninth People's Hospital (JYJC202002), and the Science and Technology Commission of Shanghai Municipality (STCSM) (22YF1422500 and JYHX2021012).

## Conflict of Interest

The authors declare that the research was conducted in the absence of any commercial or financial relationships that could be construed as a potential conflict of interest.

## Publisher's Note

All claims expressed in this article are solely those of the authors and do not necessarily represent those of their affiliated organizations, or those of the publisher, the editors and the reviewers. Any product that may be evaluated in this article, or claim that may be made by its manufacturer, is not guaranteed or endorsed by the publisher.

## Supplementary Material

Supplementary Table 1 The characteristics of the aged marmosets.

Supplementary Video The process of sevoflurane anesthesia.
